# Extralevator abdominoperineal excision for rectal cancer with biological mesh for pelvic floor reconstruction

**DOI:** 10.18632/oncotarget.12502

**Published:** 2016-10-06

**Authors:** Wei Ge, Song-song Jiang, Wang Qi, Hao Chen, Li-ming Zheng, Gang Chen

**Affiliations:** ^1^ Department of general surgery, Nanjing Drum Tower Hospital, the affiliated Hospital of Nanjing University Medical School, Nanjing, Jiangsu, P. R. China

**Keywords:** extra-levator abdominoperineal excision, ELAPE, low rectal cancer, biological mesh, pelvic reconstruction

## Abstract

**Goal:**

To share our experience of extra-levator abdominoperineal excision (ELAPE) for low rectal cancer, focusing on perineal repair with biological mesh.

**Methods:**

We retrospectively analyzed medical records of all patients with low rectal cancer who underwent the ELAPE procedure using biological mesh for perineal repair at the Gastrointestinal Surgery of Nanjing Drum Power Hospital between January 2013 and September 2015. All patients were closely followed up to now.

**Results:**

A total of 17 patients underwent ELAPE for low rectal cancer was screened. Of these, 15 patients had primary rectal cancer, 1 had local recurrent rectal cancer, and 1 had malignant melanoma. All patients underwent ELAPE successfully without intestinal perforation and got stage I healing in perineum wound without incision infection, dehiscence, cystocele perinealis, urethral dysfunction or intestinal obstruction. Perineum wound hematoma developed in just one patient and had successful percutaneous drainage in one week. During the follow-up, there was no recurrence, perineal hernia, sexual dysfunction, urinary retention, or bowel obstruction. Two patients described slight pain in the sacrococcygeal region without special handling.

**Conclusion:**

ELAPE is applicable to low rectal cancer. Biological mesh reconstruction of perineal defect seems to be safe and effective, with high patient compliance.

## INTRODUCTION

Up to now, improvements in surgical techniques for rectal cancer, with precise definition of correct surgical planes and total mesorectal excision (TME), have result in improved control of local disease and survival rate [1, 2, 3]. The incidence rate of patients with positive circumferential resection margins has dramatic declined, with also reduction of the local recurrence rate [1, 4]. However, the cancers localized in the upper and middle rectum had primarily improved, whereas low rectal cancer treated by conventional abdominoperineal excision (APE) still faces a challenge. APE led to a high local recurrence rate because of a high risk of tumor perforation and positive circumferential resection margins during operation [1, 5, 6]. Later, a method of removal of the levators has been proposed and the procedure has been named “extralevator APE” or “extra-levator abdominoperineal excision (ELAPE)”, leading to broader margins and fewer positive resection margins compared with the conventional APE [7, 8].

The ELAPE method includes removeal of the entire pelvic floor together with the anorectum. This operation produces a large defect in the pelvic floor and the perineal wound is closed by only unsubstantial skin and fat. So reconstruct the pelvic floor is needed to prevent the formation of perineal hernia. Up to now, many alternative techniques have been tried to repair the pelvic floor [9]. Primary closure is a simple method with high rates of wound complication and perineal herniation. Myocutaneous flaps that have been tried to repair the defect such as gluteus maximus[10], rectus abdominus and latissmus dorimuscles [11, 12]. Disadvantages of myocutaneous flaps include donor-site morbidity, prolonging operation time and increasing cost and resources [9]. Recently, the use of biological mesh implant has been provided to be an alternative method in pelvic floor repair and reconstruction after ELAPE [13, 14].

We introduced the ELAPE procedure and efficacy for low rectal cancer at our institution between January 2013 and September 2015, using a biological mesh for perineal repair. In this study, we report the experience of ELAPE, focusing on perineal repair with biological mesh.

## MATERIALS AND METHODS

### Patients

We retrospectively analyzed medical records of all patients with low rectal cancer who underwent the ELAPE procedure using biological mesh for perineal repair at the Gastrointestinal Surgery of Nanjing Drum Power Hospital between January 2013 and September 2015. All patients used for figures provided informed consent.

Primary tumor classification was performed by means of biopsy, digital rectal examination with evaluation of whether the tumor was mobile or fixed, measurement of the distance from the anal verge with a proctoscope, CT and MRI. None of the patients accepted preoperative neoadjuvant chemoradiotherapy. All patients were given postoperative chemotherapy.

### Extralevator abdominoperineal excision procedure

In all patients, the procedure approach was open and included three steps: abdominal operation, perineal operation and pelvic floor reconstruction. The operations were performed by the same group of surgeons.

### Abdominal operation

The abdominal operation was performed with the patient in the lithotomy position. This operation phase involved colonic and mesocolic dissection from retroperitoneal space, ligation of the inferior mesenteric artery proximal or distal to the origin of the left colic artery, and selective mobilization of the splenic flexure. Rectal dissection followed TME principles and the dissection was continued down to the anal levator starting position. Thus a complete TME was performed during the abdominal phase of the procedure. The colon was divided, a colostomy fashioned, and the abdomen closed. We did not leave a pelvic drain through the abdominal wall.

### Perineal operation

**Figure 1:**
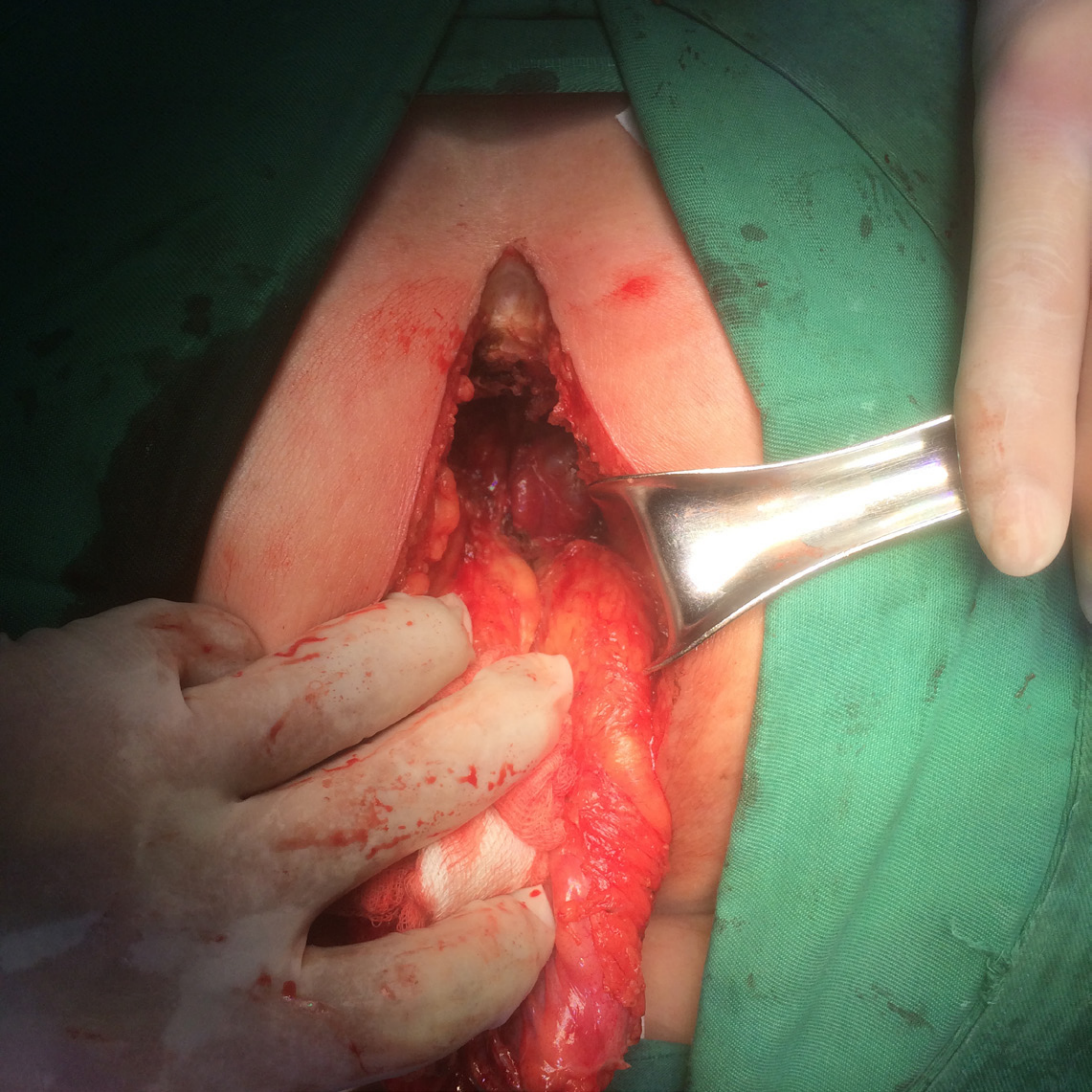

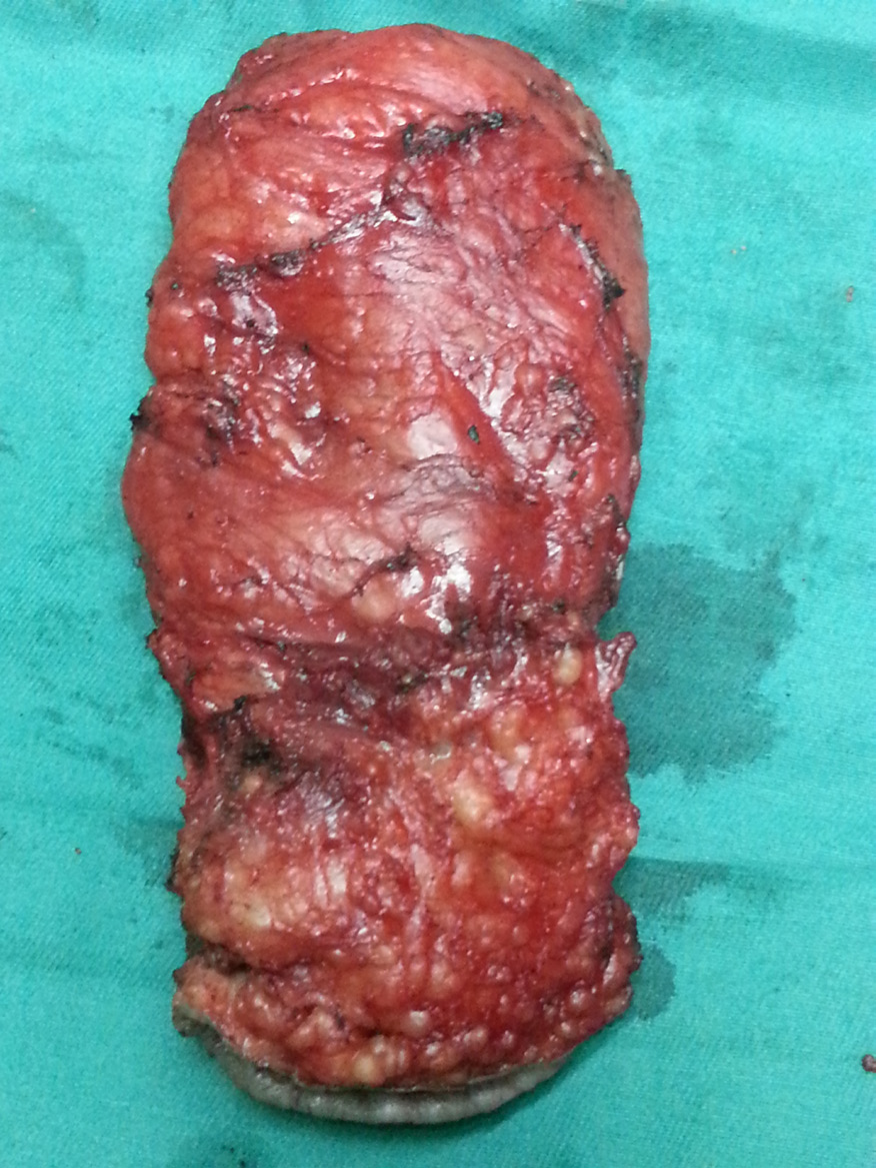
**A**. Dissect the rectum and sigmoid colon through the perineal wound. **B**. The specimen of extra-levator abdominoperineal excision.

The perineal operation was performed in the prone jack-knife position to ensure direct visualization of the anterior wall and adjacent organs. We make a fusiform incision around the anus between the bottom of the sacrum and perineum and cut open the skin and subcutaneous tissue. We excise the ischioanal fat and dissect continued laterally along the levator muscles, which were then divided close to the pelvic sidewalls (Figure 1A).. The coccyx was removed, as is routinely done to facilitate specimen retrieval and the anterior dissection continued after reflecting the specimen. Then the specimen was completely removed (Figure 1B).

### Pelvic floor reconstruction

**Figure 2:**
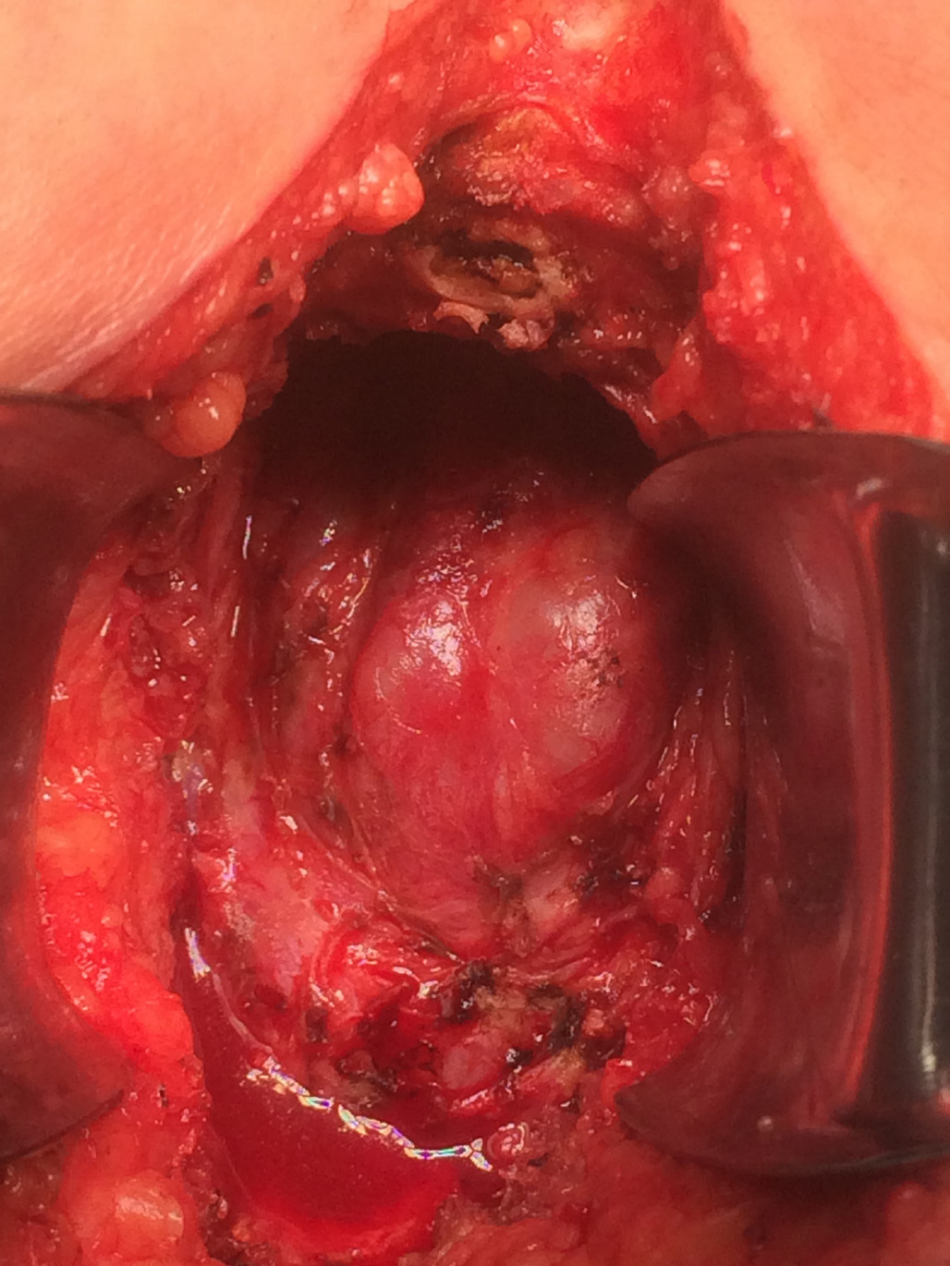

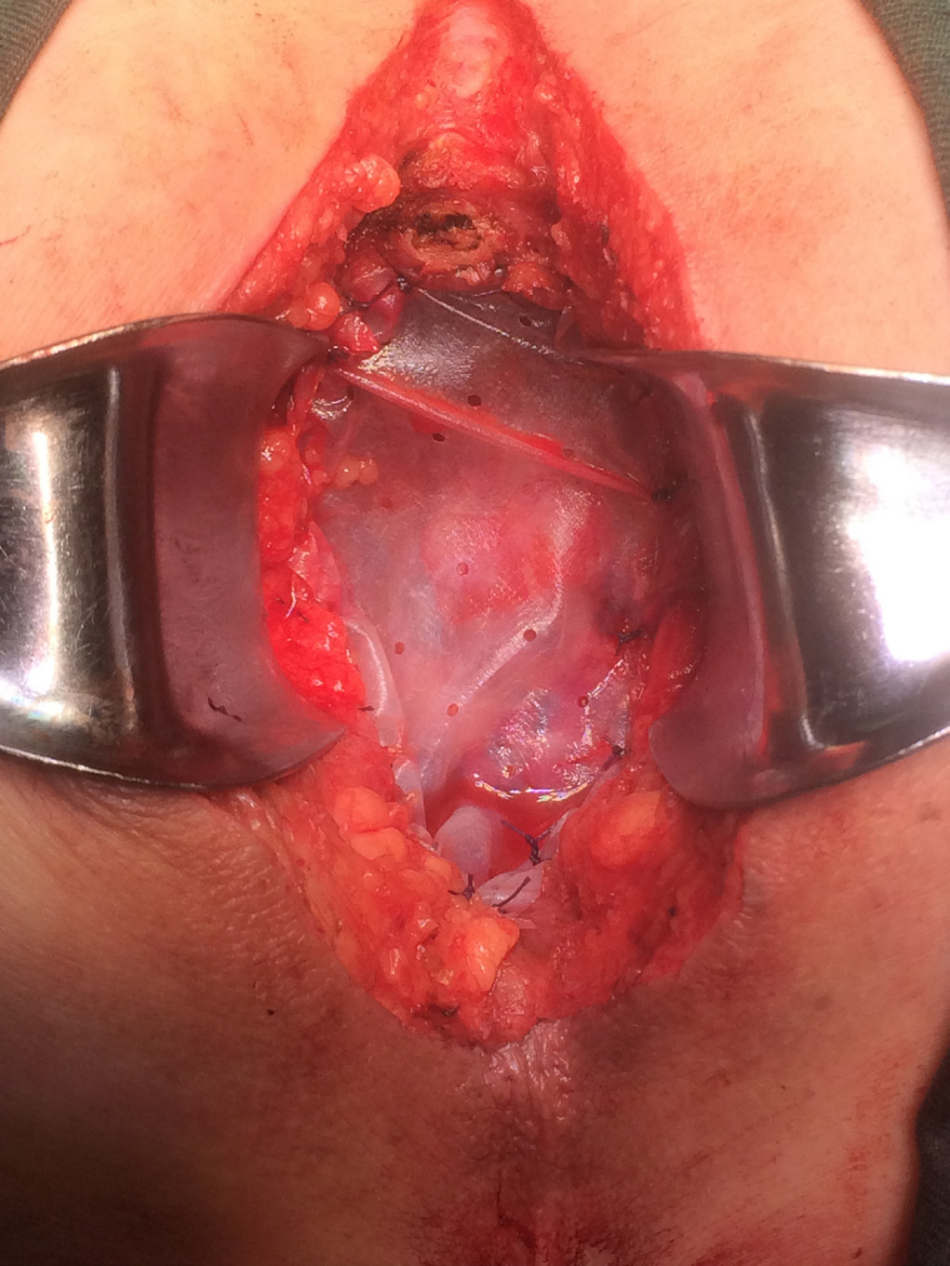
**A**. The large perineal defect following ELAPE. **B**. Pelvic floor reconstruction with Biodesign biologic mesh.

ELAPE lead to a large perineal defect (Figure 2A). In this study, we used Biodesign biologic meshes (Cook, China) for the reconstruction and the procedure involved soaking in saline solution for 5 minutes and fixation of the mesh to the cut edges of the levators by non-absorbable 2-0 sutures (Figure 2B). A perineal drain was used and kept negative pressure always, which was removed when drainage was minimal. Potassium permanganate was used for hip bath after removing the stitches.

### Stages of healing

The healing of incision is divided into three levels. I, the incision heals well without adverse effect. II, the incision heals poor with inflammation without infection. III, the incision heals bad with infection and needs drainage.

### Collection of clinical data and follow up

All patients agreed to participate in long-term follow-up including interview and clinical examination 1, 3, 6, 12 and 24 months postoperatively. All complications were registered and retrospectively collected from the medical records and interview, including perineal wound infection, patients, description of pain, movement limitations, sexual activity and impairment, as well as perineal hernia and fistula formation.

## RESULTS

### Baseline characteristics

A total of 17 patients with low rectal cancer underwent ELAPE between January 2013 and September 2015. All the clinical characteristics were summarized in Table 1. None of the patients underwent preoperative neoadjuvant chemoradiotherapy. Three patients have hypertension history, one patient have 2-diabetes mellitus history, and one patient have both hypertension and 2-diabetes mellitus history.

**Table1:** Summary of data for study sample

Demographic and pre-operative data	
Age	62.8 (39-84) years
Sex	
Male	12
Female	5
Preoperative neoadjuvant chemoradiotherapy	0
Preoperative diagnosis	
Primary rectal cancer	15
Local recurrence rectal cancer	1
Malignant melanoma	1
**Intra-operative data**	
Perforation	0
Distance from the anal edge	2.4 (0-4) cm
Maximum diameter of the tumor	3.0 (1.2-4.5) cm
**Histopathological data**	
TNM stage	
I	4
II	3
IIIA	2
IIIB	4
IIIC	3
RO resection	17
**Postoperative data**	
Incision infection	0
Incision dehiscence	0
Cystocele perinealis	0
Urethral dysfunction	0
Intestinal obstruction	0
Perineum wound hematoma	1
**Follow-up data**	
Follow up time	14 (3-30) months
Perineal hernia	0
Sexual dysfunction	0
Urinary retention	0
Bowel obstruction	0

### Operation information

Of these, 15 patients had primary rectal cancer, 1 had local recurrent rectal cancer, and 1 had malignant melanoma. All patients underwent ELAPE successfully without enterobrosis. The resection specimen was columned, consisting of middle and lower section of the rectum, low mesorectum, levator ani muscle, and anal tube. The postoperative pathologic findings showed that there were no residual neoplasms at resection margin. There were 12 cases of moderately differentiated adenocarcinoma, 4 cases of poorly differentiated adenocarcinoma, and 1 case of malignant melanoma. The TNM stage showed that there were 4 cases of stage I, 3 cases of stage II, 2 cases of IIIA, 4 cases of IIIB, 3 cases of IIIC.

### Postoperative complication

All patients got stage I healing in perineum wound without incision infection, dehiscence, cystocele perinealis, urethral dysfunction or intestinal obstruction. Perineum wound hematoma developed in just one patient and had successful percutaneous drainage in one week.

### Follow-up information

The average follow up time was 14 months (range 3-30 months). CT examination and tumor marker showed no recurrence in all patients. There was also no perineal hernia, sexual dysfunction, urinary retention, or bowel obstruction. Two patients described slight pain in the sacrococcygeal region without special handling.

## DISCUSSION

In conventional APE, inadequate resection of the lesion leads to a higher risk of positive CRM and intraoperative perforation. To resolve these problems and improve patient survival, ELAPE emerged and proven to effectively reduce CRM involvement and bowel performation[15, 16]. However, there were also researches showed that ELAPE not always reduces CRM positivity [17]. In our study, we found that there was no positive CRM or intraoperative perforation in all patients. Besides, there was also no sign of local recurrence or metastasis during the average follow-up time of 14 months. Our study suggests that patients with advanced low rectal cancer have oncological benefits from ELAPE.

Taking into account the specimen volume of ELAPE including the entire levator muscles and surrounding ischiorectal fossal fat, the defect of pelvic floor need to be rebuilt. Primary closure was the first method, which involves closing the perineal defect with the subcutaneous tissue. Its advantage was that it required minimal operative time, but disadvantage was to reconstruct the perineal defect without muscle. Primary closure might lead to high rates of wound complication and perineal herniation[18].

As myocutaneous flaps could delivery good perfusion, oxygenation and leucocyte delivery, so that they could enhance the healing process and beneficial to wounds [19]. A series of researches showed that myocutaneous flap was superior to primary closure in reducing perineal abscesses and major dehiscence [20, 21, 22]. Such methods were used as follows: gluteal flaps [23], rectus abdominis and gracilis flaps [24, 25], and so on. However, these strategies might be complicated and time-comsuming.

Later, there were evidences for the application of synthetic meshes to reconstruct the pelvic floor following ELAPE. However, this method was limited by some disadvantage s such as fistulae formation, development of small bowel adhesions and so on [26].

Recently, the application of biological mesh has provided a safe and easy alternative for pelvic floor reconstruction, thus gaining popularity [27, 28, 29]. The common used biological meshes included: human acellular dermal marx (HADM), acellular porcine small intestinal submucosa, cross-linked acellular porcine dermis and so on. Compared to myocutaneous flap reconstruction, biological meshes could simplify the perineal reconstruction, shorten operative time, and avoid flap-related complications [29]. Jensen KK et al. carried out a single-centure experience and concluded that Pelvic floor repair with a biological mesh following extralevator abdominoperineal excision produces low wound complication rates and few perineal hernias [30]. In our study, we used Biodesign (Cook, China) biological mesh, which was a non-dermis, non-cross-linked high-end tissue repair material, to reconstruct the perineal defect. This biological mesh has improved matrix technology and speed up the hydration process. So that the mesh could be metabolic absorbed without any foreign matter and patients felt comfortable when sitting and walking. Besides, this biological mesh can be used in infective environment. Our study used this biological mesh in 17 cases and all cases healed well without incision infection or dehiscence. During the follow up, there were also no phenomenons of perineal hernia, intestinal obstruction, urethral dysfunction or sexual dysfunction. Patients felt well when sitting and walking without limitation.

Chronic perineal pain was the most common postoperative complication after ELAPE. The patients might feel pain and have difficulty in sitting and walking. Han et al. found that the chronic pain maybe due to the damage to the pudendal nerve, the activation of the inflammatory cytokines and so on[31]. In our study, only two patients felt slight pain in the sacrococcygeal region with no need for special handling. We analyzed that Biodesign biological mesh could be metabolic absorbed without any foreign matter to reduce the local inflammatory reaction. So that patients felt comfortable when exercising.

## CONCLUSION

ELAPE is applicable to low rectal cancer. Biological mesh reconstruction of perineal defect seems to be safe and effective, with high patient compliance. Besides, biological mesh is technically easy to use. We believe that biological mesh for reconstruction of the pevlic floor following ELAPE will be wildly used in advanced lower rectal cancer.
